# Global Responses to Prevent, Manage, and Control Cardiovascular Diseases

**DOI:** 10.5888/pcd19.220347

**Published:** 2022-12-08

**Authors:** Fátima Coronado, Sandra Carr Melvin, Ronny A. Bell, Guixiang Zhao

**Affiliations:** 1 National Center for Chronic Disease Prevention and Health Promotion, Centers for Disease Control and Prevention, Atlanta, Georgia; 2 Institute for the Advancement of Minority Health, Ridgeland, Mississippi; 3 Department of Social Sciences and Health Policy, Wake Forest University School of Medicine, Winston-Salem, North Carolina

## Introduction

### Cardiovascular disease burden

Cardiovascular disease (CVD), a group of disorders of the heart and blood vessels that includes coronary heart disease, stroke, congestive heart failure, and other conditions, is the leading cause of death worldwide and a major contributor to disability. In 2020, an estimated 523 million people had some form of CVD, and approximately 19 million deaths were attributable to CVD; this represents approximately 32% of all global deaths and is an absolute increase of 18.7% from 2010 (1,2). Global trends for disability-adjusted life years for CVD and the CVD burden attributable to modifiable risk factors have also continued to increase steadily since 1990 (3). In the US, nearly half of adults (approximately 127 million) had 1 or more CVD condition (2). Provisional mortality data for 2021 indicate that even during the COVID-19 pandemic, heart disease and stroke remained the first and the fifth leading causes of death in the US, respectively (4). Despite advancements in the management of CVD and other health outcomes worldwide, minority, disadvantaged, and underserved populations continue to experience significant health disparities, with these disparities exacerbated during the COVID-19 pandemic (5,6). This special collection of *Preventing Chronic Disease* (PCD) highlights public health research, evaluation, and programmatic implementation that incorporate the lens of health equity to address CVD and improve the cardiovascular health of diverse populations.

### Themes of the collection

In recent years, researchers and public health programs and practices have focused on preventing, managing, and controlling traditional CVD risk factors by instituting timely intervention programs, identifying social determinants of health (SDOH), examining disparities in CVD risks, assessing the COVID-19 pandemic’s impact on CVD risks, and implementing collective efforts through community-based approaches to achieve population-level improvements in cardiovascular health. This special PCD collection of 20 articles published from January 2020 through November 2022 highlights some of these efforts by using multiple data sources collected before or during the pandemic. For instance, cigarette smoking and risk-enhancing factors related to pregnancy have been shown to increase CVD risks with significant implications (eg, increased infant mortality). Disparities in hypertension, stroke, and stroke mortality exist, exhibiting significant sociodemographic (eg, racial) and geographic (eg, rural–urban, county, zip code) variations. Intervention programs, such as behavioral modifications strengthening chronic disease awareness, use of self-measured blood pressure monitoring, and sodium intake reduction, are evaluated. The impact of COVID-19 on CVD is also explored. Finally, systematic reviews and meta-analyses evaluated the associations of circulating vitamin D levels, vitamin D supplementation, or high-density lipoprotein cholesterol (HDL-C) with blood pressure or stroke. These 20 articles advance our understanding of effective CVD risk management and intervention programs in multiple settings — in the general population and among high-risk groups — with a health equity lens across 3 broad themes further explored in this essay:

1. Examining factors contributing to CVD risk
2. Exploring factors contributing to disparities in CVD
3. Using community-based approaches to decrease CVD

## Examining the Factors Contributing to CVD Risk

The greatest contributors to CVD-related years of life lost globally are tobacco exposure, hypertension, high body mass index (BMI), and high fasting plasma glucose (3). Tobacco exposure, including cigarette smoking, secondhand smoke, and use of smokeless tobacco, contributed to 8.7 million deaths worldwide in 2019, one-third of which were due to CVD (3). Hypertension affects more than 4 billion people worldwide, representing a near doubling in the absolute prevalence of hypertension since 1990 (3). In the US, nearly half of adults (47%) have hypertension, but only about 1 in 4 (24%) have their condition under control (7). Elevated BMI continues to increase globally, with significant effects on death, disability, and quality of life (3). The prevalence of obesity has increased worldwide in the past 50 years, reaching pandemic levels. Obesity represents a major health challenge because it substantially increases the risk of diseases such as hypertension, myocardial infarction, stroke, type 2 diabetes, and dementia, thereby contributing to a decline in both quality of life and life expectancy (8). Furthermore, global increases in high fasting plasma glucose and its sequelae, type 2 diabetes, have mirrored the increases seen in BMI over the past 3 decades (9). Other behavioral risks (eg, unhealthy diet, physical inactivity, inadequate sleep, excessive alcohol use); environmental risks (eg, air pollution, extreme temperatures); and social risks (eg, house and food insecurity) also contribute to increased CVD burden and disparities in cardiovascular morbidity and mortality (10)

Several of the contextual risk factors attributed to increased CVD burden are covered in this special collection. Cigarette smoking persists among adults with chronic disease. Using data from the 2019 National Health Interview Survey (NHIS), Loretan and colleagues reported that more than 1 in 4 US adults aged 18 to 64 years with 1 or more chronic diseases associated with smoking were current smokers (11). The current cigarette smoking prevalence in the US reached 51.9% among adults aged 18 to 44 years with 2 or more chronic diseases (11). Furthermore, that study showed that smoking cessation services were not being provided to almost 1 in 3 people who have a chronic disease, leaving important steps to be taken toward successful smoking cessation in this population (11). Also concerning, rates of smoking vary significantly across countries, and approximately 1 billion people smoke globally, with significant negative implications for cardiovascular health (3). Goulding and colleagues used National Health and Nutrition Examination Survey data collected from 2011 through 2018 to provide estimates of the prevalence of high blood pressure among US children aged 8 to 17 years. The authors documented that elevated blood pressure was most prevalent among children who were older, male, or non-Hispanic Black, with factors beyond inequalities in body weight likely contributing to disparities in elevated blood pressure (12). Furthermore, a meta-analysis conducted by Qie and colleagues determined that a high level of HDL-C may provide a protective effect on the risk of total stroke and ischemic stroke but may increase the risk of intracerebral hemorrhage (13). Another meta-analysis by Zhang and colleagues found an L-shaped dose–response relationship between circulating vitamin D levels and the risk of hypertension; however, the pooled results of randomized controlled trials did not show vitamin D supplementation to be effective in preventing hypertension (14).

Studies in this collection also identified populations and communities with higher prevalence or at higher risk for CVD. In a cross-sectional study using 2018 NHIS data, Mendez and colleagues documented a higher prevalence of CVD and its risk factors among US adults with vision impairment (15). Salahuddin and colleagues documented zip code variations in infant mortality rates associated with a high prevalence of maternal cardiometabolic high-risk conditions (chronic or gestational diabetes, chronic or gestational hypertension, smoking during pregnancy, and prepregnancy obesity) in 2 counties in Texas (16). Findings from these articles could direct efforts to implement appropriate strategies to prevent, manage, and control CVD in populations at high risk.

## Exploring Factors Contributing to Disparities in CVD

CVD and its related risk factors are increasingly recognized as growing indicators of global health disparities (17). Globally, differences in morbidity and mortality from CVD exist among high-, middle-, and low-income countries and across ethnic groups (1,3,5,6,17,18). In the US, disparities in CVD morbidity, mortality, and risk factors have persisted for decades, with concerning stagnation and significant upward trends since the early 2000s (18). Disparities are largely influenced by demographic, socioeconomic, and environmental factors (19,20). For example, African American and American Indian adults experience a higher burden of cardiovascular risk factors and CVD compared with non-Hispanic White adults (18). Unfortunately, structural racism remains a significant cause of poor cardiovascular health, restricting racial and ethnic minority populations from opportunities to live healthier lives, in healthier neighborhoods, and from access to quality education and health care (20).

Several studies in this collection examine the relationship between sociodemographic characteristics, including race, ethnicity, and geography, and CVD disparities. Within this broad topic, Tong and colleagues examined data on more than 1 million Medicare fee-for-service beneficiaries aged 66 years or older hospitalized with a primary diagnosis of acute ischemic stroke (AIS). They identified significant racial, ethnic, and geographic variations in 5-year survival rates after AIS, with African American men and people living in the state of Hawaii having the lowest survival rate (21). Flynn and colleagues examined data from the National Vital Statistics System and documented marked differences in geographic patterns when using relative and absolute indicators of disparity as an appropriate measure for programs designed to decrease stroke mortality among US adults aged 35 to 64 years (22). This finding demonstrates the need to examine both measures of disparities along with race-specific rates when prioritizing efforts to eliminate racial inequities in stroke mortality (22). Furthermore, multiple factors affect the overall and cardiovascular health of rural residents (23). Hospital and outpatient facility care, clinician supply, insurance coverage, and public health infrastructure all differ between urban and rural areas, worsening disparities in CVD morbidity and mortality prominently observed among people living in rural areas (23). Tshiswaka and colleagues provide a geocoding snapshot that documents disparities in the availability of stroke centers in Florida, favoring urban counties and underscoring the need for equitable resource allocation regarding the availability of primary stroke centers in this state (24).

Available evidence suggests that influenza vaccination is associated with a protective effect in CVD morbidity and mortality (25). By using data from the Behavioral Risk Factor Surveillance System, Parekh and colleagues highlight the association of race and ethnicity and geographic location with disparities in influenza vaccination coverage among adults with CVD in the US, recommending prioritization of vulnerable populations looking beyond clinical settings as a place of vaccination (26). Compounding the challenge of seasonal influenza infection, the COVID-19 pandemic — another viral respiratory infectious disease — has exacerbated the health conditions of people with CVD worldwide and intensified disparities in CVD mortality rates in the US (6). In the US, African American, Hispanic, and Asian American populations experienced a disproportionate rise in deaths caused by heart disease and stroke, suggesting that these groups have been most impacted by the COVID-19 pandemic (6). In this collection, Tong and colleagues used data from a multistate stroke registry to examine the effect of the pandemic on stroke quality of care and demonstrated that, despite reductions in stroke hospitalizations and increased in-hospital death during the early phases of the pandemic, the adherence to quality of stroke care did not change much (27).

This collection also offers multiple recommendations and tools to identify SDOH and address disparities in CVD. For example, Le and colleagues introduce a powerful interactive visualization tool to identify county-level death rates and trends for several CVD outcomes by different sociodemographic characteristics. This online dashboard provides maps, line plots, and charts useful for health practitioners and community leaders to identify and address health inequities in CVD mortality (28). Taken together, the variations identified across different geographic, racial, or ethnic groups, as described in this theme of the collection, call for urgent actions to address disparities to understand the reasons for these variations (eg, inequities in access to care and receiving treatments). Results indicate that addressing SDOH, including equitable availability and accessibility of resources, is necessary to mitigate the factors that influence the development of CVD disparities.

## Using Community-Based Approaches to Decrease CVD Risk

Decreasing CVD risk requires strong, diverse collaborations and the implementation of innovative approaches that aim to eliminate health disparities and advance health equity in diverse environments and contexts. Eliminating health disparities and advancing health equity should be core components in all research, evaluation, and programmatic activities and require a focus on SDOH (29). Systematically addressing SDOH requires multisectoral commitment and the implementation of evidence-based public policies and actions across all sectors. Countries that employ multisectoral approaches are better able to identify and address issues around poverty, housing, and others by working collaboratively across sectors, with multisectoral action by governments to achieve health equity (30).

Several studies in this collection identify strategies for community-based interventions that aim to reduce CVD disparities. For example, Long and colleagues evaluated 3-year sodium reduction initiatives in 3 community meal programs in Arkansas (31). Jordan and colleagues demonstrated the differential effects of sodium reduction strategies in food service settings by tailoring community-level approaches based on a community’s available resources, stage of readiness, and food service staff’s level of engagement (32). These studies show the effectiveness and sustainability of the implementation of sodium reduction interventions in reducing CVD in communities experiencing food insecurity, low incomes, and high risk for hypertension (31,32). The work from Smith and colleagues in Arkansas examines the benefits of using trusted community spaces such as barber and beauty shops for screening for chronic health conditions including blood pressure monitoring. Their findings indicate that community-based settings are effective in increasing knowledge of CVD-related risk factors and access to health promotion resources to reach minority populations (33).

Furthermore, both Stupplebeen and Sreedhara and their colleagues explored their experiences in implementing self-measured blood pressure monitoring and telehealth to address hypertension (34,35). Readers can draw from their experiences to make improvements to their hypertension control programs and initiatives. Finally, Stanhope and colleagues conducted qualitative interviews in the midst of the pandemic with postpartum patients who had a hypertensive disorder of pregnancy. Their work elaborates on the need to improve the uptake of preventive behaviors among postpartum patients at risk for heart disease through continuity and content of care improvements (36).

Collaborative innovations are beneficial to prevent, manage, and control CVD and risk factors. For example, as described by Abbas and colleagues, several clinicians and health care organizations were able to accelerate innovation and adapt services to maintain hypertension control among their high-risk populations during the COVID-19 pandemic, informing future collaborative efforts related to hypertension control during and after a public health emergency (37). Furthermore, as highlighted by Ramalingam and colleagues from their work in India, there is a need to invigorate and transform the public health workforce to prevent and control noncommunicable diseases. They do so through the innovative Field Epidemiology Training Program in noncommunicable diseases, which enhances workforce capacity in CVD epidemiology, surveillance, and evaluation to inform CVD control programs and policies. For instance, in India, resident projects focus on investigating aspects of hypertension epidemiology and management in collaboration with local partners (38). These types of community-based approaches can help to transform the social and environmental conditions affecting traditionally marginalized populations affected by CVD.

## Summary of Key Findings

The authors in this collection share lessons learned that represent experiences in diverse aspects of CVD prevention, management, and control. Their work highlights the multiple contextual health-related behaviors and cardiometabolic risk factors attributed to increased CVD burden. Studies in the collection discussed prevention strategies to optimize health behaviors to reduce the development of CVD risk factors or to avert the development or progression of disease. This collection also takes a view of pervasive disparities in the prevention and control of CVD and underscores the challenge and need to reposition evidence-based strategies to confront disparities.

Strategies described in this special collection such as telemedicine, engaging patients in self-measured blood pressure monitoring, adapting or implementing medication management services, activating partnerships, expanding services to respond to patient needs, and implementing unique patient outreach approaches also proved promising. Furthermore, tools and resources presented in this collection can be adapted to identify and address SDOH through tailored strategies, programs, and policies that can address the needs of populations disproportionately affected by CVD.

Nevertheless, much work remains to be done to address other factors contributing to CVD beyond those presented in this special collection, including the reasons for identified disparities in CVD and specific strategies to confront them, and to explore the intertwined effects of traditional risk factors, health care access, and SDOH on CVD risk and risk reduction. To address SDOH, efforts may need to be directed toward improving data systems to systematically measure SDOH, including racism and the social and psychological determinants affecting populations at higher risk or with higher incidence of disease, in a timely, relevant, and actionable manner. In addition, a major gap identified among articles appearing in the collection is the lack of focus, research questions, or emphasis on the impact of racism on cardiovascular health. Evidence has shown the significant impact of structural racism on poor health and premature death due to heart disease and stroke (20,39,40). Other areas not explored in this collection that deserve further examination include the lack of evidence on the long-term impact of COVID infection on the risk and burden of CVD, effects of COVID vaccinations in CVD management, and assessments of cardiovascular health globally.

## Implications

The articles in this collection reflect the magnitude of CVD and its risk factors across the globe. Preventing, managing, and controlling CVD will require the collective effort of policy and decision makers, clinical and public health practitioners, and researchers. Cardiovascular health may be improved by focusing on decreasing disparities in CVD, advancing health equity, and addressing SDOH. This collection of articles suggests that evidence-based and multicomponent interventions are necessary to address inequities and advance health equity. Furthermore, findings from this collection can be used to guide the development of community-based interventions to reduce cardiovascular disparities that are culturally appropriate, with a focus on health equity. Future research and evaluation of programs should focus on developing practical and innovative strategies, identifying and overcoming the barriers to access to quality care, and applying a health equity lens to accelerate advances in CVD prevention and control at the community, state, national, and global levels.
